# Change in Kidney Function and 2-Year Mortality After Transcatheter Aortic Valve Replacement

**DOI:** 10.1001/jamanetworkopen.2021.3296

**Published:** 2021-03-26

**Authors:** Guy Witberg, Tali Steinmetz, Uri Landes, Rotem Pistiner Hanit, Hefziba Green, Shira Goldman, Hana Vaknin-Assa, Pablo Codner, Leor Perl, Benaya Rozen-Zvi, Ran Kornowski

**Affiliations:** 1 Department of Cardiology, Rabin Medical Center–Beilinson Hospital, Petach Tikva, Israel; 2 Sackler School of Medicine, Tel Aviv University, Tel Aviv, Israel; 3 Department of Nephrology, Rabin Medical Center–Beilinson Hospital, Petach Tikva, Israel

## Abstract

**Importance:**

Chronic kidney disease (CKD) is prevalent in the population of patients undergoing transcatheter aortic valve replacement (TAVR). Data on the association of TAVR with kidney function are scarce, as are data on the relationship between changes in kidney function after TAVR and mortality.

**Objective:**

To describe the changes in kidney function (both periprocedural and at steady state) after TAVR and to explore the association of TAVR with midterm mortality.

**Design, Setting, and Participants:**

This single-center, retrospective cohort study was conducted at a public, tertiary academic medical center, which serves as a regional referral center for valvular heart interventions. Consecutive cases of patients undergoing TAVR from November 5, 2008, to December 31, 2019, were included in the study, with available baseline and post-TAVR data on kidney function.

**Exposures:**

Steady state (1 month) change in kidney function after TAVR. Significant improvement or deterioration in renal function was defined as a greater than or equal to 10% change in estimated glomerular filtration rate (eGFR).

**Main Outcomes and Measures:**

Overall mortality at 2-year follow-up.

**Results:**

A total of 894 patients (mean [SD] age, 82.2 [7.1] years; 452 women ([51.2%]) were evaluated. A total of 362 patients (40.5%) were treated from 2017 to 2019, 348 patients (38.9%) were treated from 2013 to 2016, and 184 patients (20.5%) were treated from 2008 and 2012. Patients had a mean (SD) Society of Thoracic Surgeons (STS) score of 5.2% (4.0%) and a mean (SD) eGFR of 65.1 (23.1) mL/min/1.73 m^2^. Acute kidney injury occurred in 115 (11.1%) patients by 48 hours, of whom 73 (63.5%) resolved by discharge. One month after TAVR, eGFR improved by at least 10% in 329 patients (36.8%) and deteriorated by at least 10% in 233 patients (26.1%). Overall, CKD stage remained stable or improved in 720 patients (80.6%), and only 5 patients (0.97%) progressed to stage 5 CKD 1 month after TAVR. A deterioration of 10% or greater in eGFR 1 month after TAVR was associated with a hazard ratio of 2.16 (95% CI, 1.24-5.24; *P* = .04) at 2-year mortality. Patients who showed CKD status resolution (eGFR improvement to >60 mL/min/1.73 m^2^ after TAVR) had a similar 2-year mortality to those with baseline eGFR greater than 60 mL/min/1.73 m^2^ and vice versa. Factors associated with steady state CKD status resolution after TAVR included lower STS score, higher left ventricular ejection fraction, higher baseline eGFR, no acute kidney injury at discharge from the TAVR admission, and lower contrast-eGFR ratio.

**Conclusions and Relevance:**

In this cohort study, kidney outcomes after TAVR were reassuring; greater than 80% of patients showed stable or improved kidney function 1 month after the procedure. Improvement in kidney function was associated with a lower 2-year mortality, whereas deterioration in kidney function was associated with increased mortality. Our data suggest that cardiorenal syndrome was a possible cause of CKD in patients in need of TAVR and that there was potential for improvement in both renal and cardiac function after this procedure.

## Key Points

**Question:**

Is transcatheter aortic valve replacement (TAVR) associated with changes in kidney function or midterm mortality?

**Findings:**

In a cohort study of 894 patients who underwent TAVR, kidney function improved or remained stable in 80.6% (improved in 36.8%) of patients 1 month after TAVR. Steady state kidney function had a stronger association with mortality than did baseline kidney function.

**Meaning:**

Results suggest that overall, TAVR is safe in terms of kidney outcomes and has the potential to improve kidney function and provide an additive benefit to its cardiac benefits.

## Teaser

This cohort study uses data from a tertiary academic medical center to assess whether transcatheter aortic valve replacement is associated with changes in kidney function or mortality in patients undergoing the procedure.

## Introduction

Transcatheter aortic valve replacement (TAVR) is now established as the preferred treatment for older adults with severe aortic stenosis (AS) who are at intermediate or higher surgical risk (when femoral access is feasible).^1^ The population of patients with TAVR is characterized by a high prevalence of moderate or worse chronic kidney disease (CKD),^2^ which is associated with increased mortality after TAVR.^2,3^ The effect of TAVR on kidney function is complicated: on one hand, multiple aspects related to the procedure (eg, exposure to contrast, atheroembolic disease, hypotensive episodes, bleeding) can result in acute kidney injury (AKI) in up to 50% of TAVR cases.^4^ Patients with baseline kidney dysfunction are at the highest risk for AKI, which is associated with a significant increase in the risk for short- and intermediate-term mortality.^4^ On the other hand, it can be hypothesized that the hemodynamic benefits of successfully treating AS, such as improved cardiac output, reduced vascular tone, better kidney perfusion, and reduced venous pressure, have the potential to improve kidney function, similar to the effect of improvement in cardiac function in patients with type 2 cardiorenal syndrome (CRS).^5^ We previously reported that for patients with moderate or worse CKD who suffer from AS, TAVR is associated with a stabilization of kidney function, whereas conservative treatment is associated with deterioration in kidney function.^6^ Data from the Placement of Aortic Transcatheter Valves 1 (PARTNER 1) trial and registry of patients with high or inoperable AS undergoing TAVR reported that for such patients (baseline estimated glomerular filtration rate [eGFR] ≤60 mL/min/1.73 m^2^), TAVR using a balloon-expandable valve was associated with 10% or greater improvement in eGFR 30 days postprocedure in 42% of treated patients and a deterioration of 10% or greater in 24% of patients. Those with kidney function deterioration had a 50% higher risk for 1-year mortality.^7^ Combined data from the PARTNER 1 and PARTNER 2 trials and registry recently reported that in patients at intermediate or high surgical risk who undergo TAVR with a balloon-expandable valve, CKD stage remained stable or improved in 89% of patients 7 days after TAVR, and the risk for progression to stage 5 CKD was negligible (0.035%).^8^ Whether the same association is true for the overall patient population not enrolled in clinical trials, patients at lower risk (who represent the majority of patients undergoing TAVR in current clinical practice^9^), and those undergoing TAVR with self-expandable valves has not been examined. Because a substantial fraction of TAVR-related AKI cases are reversible and given the results of the PARTNER 1 trial and registry mentioned previously (which indicate that many patients may experience an improvement in kidney function after TAVR), it is likely the steady state kidney function after TAVR, rather than baseline or periprocedural kidney function, that has the more prognostic association with mortality. Our goal in this study was to examine the association of TAVR with kidney function (both periprocedural and steady state) and the association between kidney function change after TAVR and 2-year mortality in a large, single-center cohort.

## Methods

This was a single-center, retrospective cohort study. Data were collected for all patients who underwent TAVR between November 5, 2008, and December 31, 2019, at Rabin Medical Center, Petach Tikva, Israel, a tertiary, academic, public medical center. The study was approved by our institutional review board, and a waiver from informed consent was granted owing to its retrospective nature. This study followed the Strengthening the Reporting of Observational Studies in Epidemiology (STROBE) reporting guideline.

### Patient Population

We reviewed our institution’s TAVR registry and calculated the baseline (<2 days before TAVR) and 1-month post-TAVR eGFR for each patient using the Chronic Kidney Disease Epidemiology Collaboration (CKD-EPI) formula^10^ and the serum creatinine (SCr) measurements from the patients’ electronic medical records. The medical records contained all the laboratory results taken either during admission or in the community setting. Patients were excluded if they required chronic hemodialysis during the year before TAVR, did not survive 1 month after TAVR, were still admitted to the hospital 1 month after TAVR, or did not have an SCr measurement 1 month after TAVR . Patients were excluded from the analysis if they did not survive to hospital discharge or did not have an SCr measurement available in the 48 hours after TAVR and at discharge.

Data on baseline characteristics (demographic details, comorbidities, and echocardiographic data), procedural characteristics, and follow-up were obtained from our institutional TAVR registry. Frailty was defined using the Rockwood Clinical Frailty Scale.^11^

In our practice, we do not perform percutaneous coronary interventions (PCI) concomitantly with TAVR in order to minimize contrast exposure. When significant coronary disease with a clear indication for revascularization is discovered during the workup for TAVR, patients are scheduled for elective PCI, usually at least 2 weeks before the scheduled TAVR. Transcatheter aortic valve replacement is performed only after reassessment of kidney function to determine that it has not deteriorated after PCI.

For all included patients, we calculated the eGFR and CKD status at baseline, 48 hours after TAVR, at discharge from the TAVR admission, and 1 month after TAVR.

Periprocedural AKI was based on a modified version of the Valve Academic Research Consortium 2 (VARC 2) criteria^12^ using the highest SCr measurement (without assessing urine output) during the 48 hours after TAVR (not during the 7 days after TAVR as defined by the VARC 2 criteria, because the median admission length in our cohort was 2 days). However, if patients developed AKI during the 48 hours after TAVR, follow-up of SCr level was continued (either in the hospital or the outpatient setting) until stabilization of kidney function as required in the VARC 2 criteria. Patients were defined as having CKD if eGFR was less than 60 mL/min/1.73 m^2^ or not having a diagnosis of CKD if eGFR was at least 60 mL/min/1.73 m^2^.

Periprocedural AKI was defined as no AKI, AKI resolved (AKI during the 48 hours after TAVR that resolved by discharge), or AKI persistent (AKI during the 48 hours after TAVR that did not resolve by discharge). Steady state changes in eGFR after TAVR were defined as kidney function improvement after TAVR (an increase of 10% or more in eGFR 1 month after TAVR compared with baseline), kidney function deterioration after TAVR (a decrease of 10% or more in eGFR 1 month after TAVR compared with baseline), or kidney function stable after TAVR (eGFR within 10% of baseline value 1 month after TAVR). Steady state CKD status changes after TAVR were defined as CKD resolution (eGFR <60 mL/min/1.73 m^2^ at baseline and eGFR ≥60 mL/min/1.73 m^2^ 1 month after TAVR) or new CKD (eGFR ≥60 mL/min/1.73 m^2^ at baseline and eGFR <60 mL/min/1.73 m^2^ 1 month after TAVR). The primary end point was 2-year all-cause mortality.

### Statistical Analysis

Patients were grouped into 3 subgroups according to the steady state changes in eGFR after TAVR (improvement, deterioration, stable). Baseline characteristics were compared between the groups using analysis of variance for continuous variables and the χ^2^ test and Fisher exact test for categorical variables, as appropriate. To identify factors associated with change in kidney function, we fitted a logistic regression model with the kidney outcome (AKI, AKI resolution, kidney function deterioration, kidney function improvement, CKD resolution, or new CKD) as the dependent variable. All baseline variables were considered for inclusion in the multivariate models based on their relevance and their pathophysiologic connection with the examined outcome. To examine the association of change in kidney function with 2-year mortality, we fitted a Cox proportional hazard ratio (HR) model that included 2-year mortality as the outcome and all relevant baseline characteristics in addition to periprocedural or steady state changes in eGFR function as covariates. For all analyses, *P* values were 2-sided, and *P* < .05 was considered statistically significant. All analyses were performed using SPSS, version 25 software (IBM Corp).

## Results

A total of 1038 of 1084 (95.8%) patients treated at our center between January 1, 2008, and December 31, 2019, were included in the study; reasons for patient exclusion are shown in eFigure 1 in the Supplement.

Serum creatinine levels at 1 month after TAVR were available for 894 patients (mean [SD] age, 82.2 [7.1] years; 452 [51.2%] women). A total of 362 patients (40.5%) were treated from 2017 to 2019, 348 patients (38.9%) were treated from 2013 to 2016, and 184 patients (20.5%) were treated from 2008 and 2012. Patients had a mean (SD) Society of Thoracic Surgeons (STS) score of 5.2% (4.0%) and a mean (SD) eGFR of 65.1 (23.1) mL/min/1.73 m^2^. Baseline characteristics of patients according to steady state change in kidney function after TAVR are shown in Table 1. Patients whose kidney function improved after TAVR had lower baseline kidney function compared with stable GFR and GFR deterioration (mean eGFR [SD] mL/min/1.73 m^2^, 57.1 [19.7] vs 69.0 [22.6] and 68.9 [28.7], either as a continuous or a categorical variable, respectively; *P* = .007 for both). Patients whose kidney function deteriorated after TAVR had a higher contrast-eGFR ratio during TAVR than those with stable GFR and GFR improvement (mean [SD] contrast-GFR ratio, 3.3 [3.8] vs 2.5 [1.8] and 3.1 [3.6], respectively; *P* = .01) and were more likely to experience AKI that did not resolve before discharge from the TAVR admission compared with stable GFR and GFR improvement (36 patients [15.6%] vs 14 [4.3] and 9 [2.8%], respectively; *P* = .005). All other baseline characteristics were similar between the 3 groups. There was an inverse association between baseline eGFR and the percent change in eGFR at 1 month after TAVR (*R^2^* = –0.052) (eFigure 2 in the Supplement).

**Table 1.** Baseline Characteristics According to eGFR Change 1 Month After TAVR

Characteristic	No. (%)^a^			*P* value
	Stable eGFR (n = 332)	eGFR improvement (n = 329)	eGFR deterioration (n = 233)	
Demographic characteristics and comorbidities				
Age, mean (SD), y	82.1 (6.6)	81.9 (7.6)	82.5 (7.3)	.58
BMI, mean (SD)	27.8 (5.0)	28.2 (5.4)	27.8 (4.9)	.54
Women	166 (50.0)	177 (53.8)	109 (46.8)	.25
Men	166 (50.0)	145 (44.1)	124 (53.2)	
NYHA class III/IV	253 (76.1)	252 (76.5)	191 (82.0)	.19
Previous PCI	115 (34.5)	116 (35.3)	81 (34.7)	.98
Previous MI	48 (14.6)	41 (12.6)	20 (8.7)	.15
Frailty^b^	43 (12.8)	47 (14.3)	32 (13.7)	.84
AF	63 (19.0)	63 (19.0)	50 (21.5)	.74
Previous pacemaker	26 (7.8)	27 (8.2)	21 (9.0)	.88
COPD	46 (14.0)	54 (16.5)	49 (21.1)	.08
Diabetes	143 (43.1)	127 (38.7)	89 (38.2)	.40
Hypertension	312 (93.9)	304 (92.4)	217 (93.1)	.74
STS score, mean (SD), %	4.7 (3.7)	5.1 (3.9)	5.8 (4.5)	.004
Laboratory results				
Hb, mean (SD), g/dL^c^	12.1 (1.7)	11.7 (1.7)	11.9 (2.0)	.31
eGFR, mean (SD), mL/min/1.73 m^2^	69.0 (22.6)	57.1 (19.7)	68.9 (28.7)	.007
eGFR <60 mL/min/1.73 m^2^, %	116 (34.9)	156 (47.5)	91 (39.1)	.006
Echocardiographic parameters, mean (SD)				
AVG peak, mm Hg	76.2 (21.9)	76.0 (24.1)	76.5 (22.2)	.97
AVG mean, mm Hg	47.8 (15.5)	48.0 (16.6)	48.5 (15.2)	.88
AVA, cm^2^	0.67 (0.18)	0.66 (0.18)	0.67 (0.18)	.70
LVEF, %	56.1 (8.7)	56.6 (8.1)	55.7 (9.4)	.47
Procedural characteristics				
Contrast, mean (SD), mL	151.9 (56.1)	148.5 (61.9)	164.1 (70.4)	.02
Contrast to eGFR ratio, mean (SD)	2.5 (1.8)	3.1 (3.6)	3.3 (3.8)	.01
Contrast to eGFR ratio >3.5	53 (15.8)	75 (22.7)	53 (22.8)	.07
Self-expandable valve	244 (73.5)	252 (76.6)	169 (72.5)	.49
Year of procedure				
2008-2012	64 (19.3)	63 (19.2)	55 (23.5)	.61
2013-2016	134 (40.4)	124 (37.8)	90 (38.7)	
2017-2019	133 (40.3)	141 (43.0)	88 (37.8)	
Postprocedural data				
AKI at discharge	14 (4.3)	9 (2.8)	36 (15.6)	.005
AKI 48 h after TAVR	23 (7.0)	20 (6.2)	57 (24.6)	<.001
Length of admission, median, d	2 (2-3)	2 (2-3)	4 (3-6)	.003

Abbreviations: AF, atrial fibrillation; AKI, acute kidney injury; AVA, aortic valve area; AVG, aortic valve gradient; BMI, body mass index (calculated as weight in kilograms divided by height in meters squared); COPD, chronic obstructive pulmonary disease; eGFR, estimated glomerular filtration rate; Hb, hemoglobin; LVEF, left ventricular ejection fraction; MI, myocardial infarction; NYHA, New York Heart Association; PCI, percutaneous coronary intervention; STS, Society of Thoracic Surgeons; TAVR, transcatheter aortic valve replacement.

^a^ Values are presented as No. (%) unless otherwise specified.

^b^ According to the Rockwood Clinical Frailty Scale, range 0-1, with higher values representing more severe frailty.

^c^ To convert g/dL to g/L, multiply by 10.

Periprocedural AKI occurred in 115 patients (11.1%; 59 [51.3%] in stage 1 and 56 [48.7%] in stage 2) within 48 hours after TAVR; of these, 73 (63.5%) had their cases resolved by discharge. According to steady state SCr measurement 1 month after TAVR, 332 patients (37.1%) showed stable kidney function; 329 (36.8%), improved kidney function; and 233 (26.1%), deterioration in kidney function. When comparing CKD stage at baseline vs 1 month after TAVR, the overall likelihood of CKD stage remaining stable or improving was 80.6% (n = 720; 58.9%, 83.8%, 82.2%, 87.3%, and 81.0% for patients with baseline CKD stages 1, 2, 3a, 3b, and 4, respectively). Progression to CKD stage 5 occurred in 5 (0.97%) patients (Table 2).

**Table 2.** Change in CKD Stage From Baseline to 1 Month After TAVR

Stage	No.	No. (%)		
		Stable	Improved	Deteriorated
CKD 1 (n = 140)				
1	66	66 (58.9)	0	45 (41.1)
2	41			
3a	3			
3b	1			
4	0			
5	1			
No data	28			
CKD 2 (n = 460)				
1	69	256 (65.9)	69 (17.8)	63 (16.3)
2	256			
3a	53			
3b	8			
4	2			
5	0			
No data	72			
CKD 3a (n = 257)				
1	6	98 (44.5)	83 (37.7)	39 (17.8)
2	77			
3a	98			
3b	37			
4	0			
5	2			
No data	37			
CKD 3b (n = 132)				
1	0	55 (46.6)	48 (40.6)	15 (12.8)
2	12			
3a	36			
3b	55			
4	15			
5	0			
No data	14			
CKD 4 (n = 47)				
1	1	17 (45.9)	15 (37.8)	5 (13.6)
2	1			
3a	3			
3b	10			
4	17			
5	5			
No data	10			
CKD 5 (n = 19)				
1	0	18 (94.7)	1 (5.3)	0
2	0			
3a	0			
3b	1			
4	0			
5	18			
No data	0			

Abbreviations: CKD, chronic kidney disease; TAVR, transcatheter aortic valve replacement.

The distribution of steady state change in kidney function after TAVR is shown in eFigure 3 in the Supplement; the mean (SD) percentage change was 4.9% (1.6%). According to steady state kidney function after TAVR, CKD status changed in 164 patients (38%): 97 of 394 patients (24.6%) with a baseline eGFR less than 60 mL/min/1.73 m^2^ had an eGFR greater than or equal to 60 mL/min/1.73 m^2^, and 67 of 500 patients (13.4%) with a baseline eGFR of greater than or equal to 60 mL/min/1.73 m^2^ had an eGFR less than 60 mL/min/1.73 m^2^ (eFigure 4 in the Supplement).

### Association Between Periprocedural Complications and Periprocedural AKI and Steady State eGFR Function

The rates of periprocedural vascular and bleeding complications were higher in patients who developed AKI (major vascular complications, 14 of 115 patients [12.1%] with AKI vs 15 of 921 patients [1.6%] without AKI; life-threatening bleeding, 3 of 115 patients [2.6%] with AKI vs 1 of 921 patients [0.1%] without AKI), whereas the need for pacemaker implantation was similar between patients with or without AKI (20 of 115 [17.3%] vs 111 of 921 [12.1%], respectively). There was no difference in vascular complications, bleeding complications, or the need for pacemaker implantation between patients with steady state stable, improved, or deteriorated kidney function (eTables 1 and 2 in the Supplement).

### Association of AKI With 2-Year Mortality

**Figure 1.**
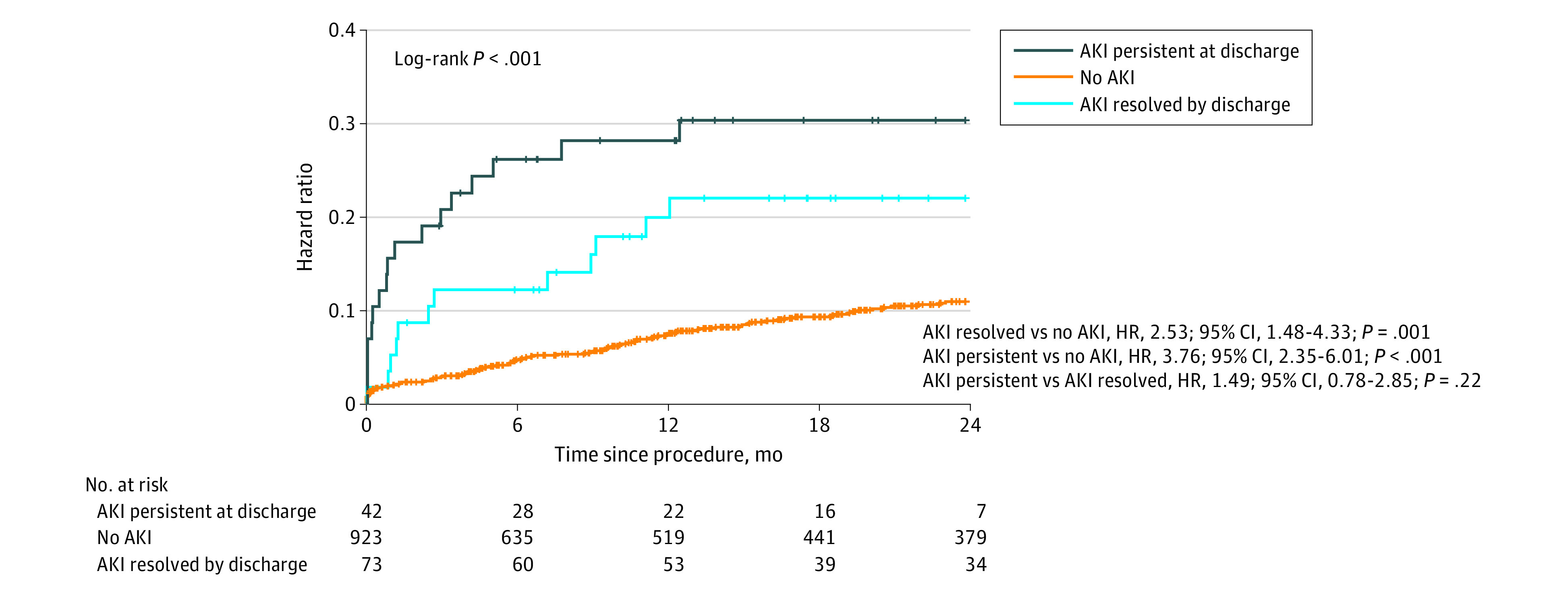
Kaplan-Meier Curves for 2-Year Mortality According to Acute Kidney Injury (AKI) Status After Transcatheter Aortic Valve Replacement (TAVR) HR indicates hazard ratio.

Acute kidney injury had an HR of 3.76 (95% CI, 2.35-6.01; *P* < .001) for 2-year mortality compared with those discharged without AKI. Those with AKI at 48 hours that resolved by discharge still showed an HR of 2.53 (95% CI, 1.48-4.33; *P* = .001) for mortality compared with patients who did not develop AKI (Figure 1).

### Association of Steady State eGFR Change With 2-Year Mortality

**Figure 2.**
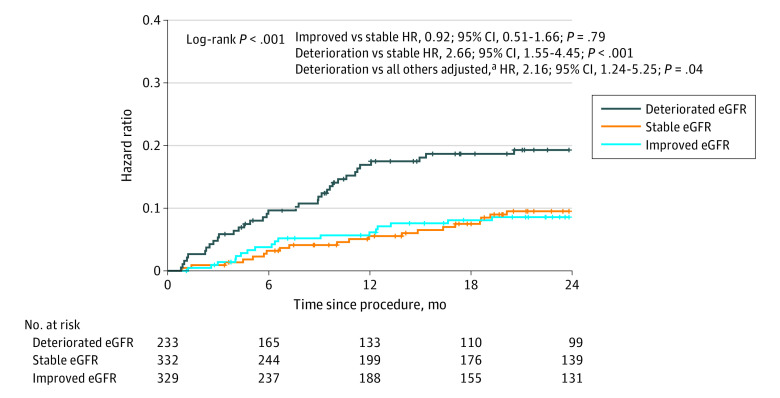
Kaplan-Meier Curves for 2-Year Mortality According to Steady State Kidney Function Change After Transcatheter Aortic Valve Replacement eGFR indicates estimated glomerular filtration rate; HR, hazard ratio. ^a^Adjusted for baseline chronic kidney disease and Society of Thoracic Surgeons score.

Patients whose steady state kidney function improved after TAVR had similar mortality to those with stable kidney function (HR, 0.92; 95% CI, 0.51-1.66; *P* = .79). However, deterioration in steady state kidney function was associated with increased mortality compared with those with either stable or improved renal function (HR, 2.66; 95% CI, 1.55-4.45; *P* < .001 and HR, 2.73; 95% CI, 1.35-3.98; *P* < .001, respectively). After multivariate adjustment, the HR for mortality for patients with steady state deterioration in renal function after TAVR was 2.16 (95% CI, 1.24-5.25; *P* = .04) (Figure 2). When analyzed as a continuous variable, each 10% decrease in steady state eGFR was associated with a 19.3% (95% CI, 16.8%-21.8%) increased risk for 2-year mortality (*P* < .001). The increased mortality risk for patients with deterioration in steady state kidney function after TAVR was associated with increased mortality in patients with baseline eGFR above or below 60 mL/min/1.73 m^2^ (eFigure 5 in the Supplement).

### Association of CKD Status Change Following TAVR With 2-Year Mortality

**Figure 3.**
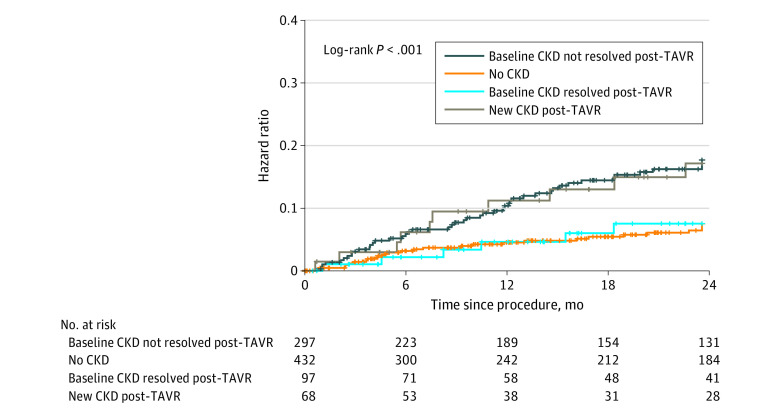
Kaplan-Meier Curves for 2-Year Mortality According to Steady State Change in Chronic Kidney Disease (CKD) Status After Transcatheter Aortic Valve Replacement (TAVR)

When analyzing survival according to CKD status before and after TAVR, patients whose CKD status improved after TAVR had similar survival compared with those with no CKD at baseline. Those with new CKD 1 month after TAVR showed similar mortality to those with CKD at baseline (2-year mortality, 7.5% [95% CI, 1.6%-13.4%]; 7.5% [95% CI, 4.8%-10.2%]; 17.1% [95% CI, 7.3%-26.9%]; and 17.6% [12.9%-22.3%], respectively) (Figure 3).

### Periprocedural and Steady-State Changes in eGFR After TAVR

In patients who developed AKI within 48 hours after TAVR, independent factors associated with AKI resolution before discharge were lack of frailty (odds ratio [OR], 4.77; 95% CI, 1.44-15.79; *P* = .01) and age younger than 80 years (OR, 8.20; 95% CI, 2.70-14.64; *P* < .001) (eTable 3 in the Supplement). Factors associated with steady state kidney function deterioration were higher STS score (STS >6%, OR, 1.43; 95% CI, 1.03-1.98; *P* = .03), higher baseline eGFR (GFR >75 mL/min/1.73 m^2^, OR, 1.92; 95% CI, 1.40-2.62; *P* = .0013, and higher AKI during 48 hours after TAVR (OR, 2.82; 95% CI, 1.70-4.70; *P* < .001) (with a stronger association if not resolved by discharge; OR, 7.32; 95% CI, 4.53-11.42; *P* < .001). Factors associated with steady state kidney function improvement after TAVR were female sex (OR, 1.27; 95% CI, 1.03-1.61; *P* = .04) and eGFR less than 60 mL/min/1.73 m^2^ (OR, 2.28; 95% CI, 1.73-3.01; *P* = .004) (eTable 3B and 3C in the Supplement). A model based on these variables had a C statistic of 0.674 (95% CI, 0.631-0.716; *P* < .001) for estimating steady state kidney function deterioration after TAVR (eFigure 6 in the Supplement).

For patients with CKD at baseline, factors associated with steady state CKD status resolution after TAVR were lower STS score (STS <6%, OR, 1.81; 95% CI, 1.10-2.97; *P* = .02), higher LVEF (LVEF >50%, OR, 2.15; 95% CI, 1.11-4.18; *P* = .02), higher baseline eGFR (eGFR >45 mL/min/1.73 m^2^, OR, 3.13; 95% CI, 1.83-6.92; *P* = .003), no AKI at discharge from the TAVR admission (OR, 4.62; 95% CI, 2.15-6.44; *P* < .001), and lower contrast-eGFR ratio (ratio <3.5; OR, 4.03; 95% CI, 2.11-7.69; *P* < .001) (eTable 3D in the Supplement). A model based on these variables had a C statistic of 0.700 (95% CI, 0.637-0.764; *P* < .001) for estimating steady state CKD after TAVR (eFigure 6 in the Supplement).

## Discussion

In this study, we assessed the periprocedural and steady state changes in kidney function after TAVR and examined their association with 2-year mortality in the overall (rather than the CKD-only) TAVR population. Our main results suggest that (1) most periprocedural AKI resolved before discharge from the TAVR admission, (2) steady state eGFR improved by 10% or more in approximately one-third of patients and deteriorated by 10% or more in approximately one-quarter of patients, (3) CKD stage remained stable or improved in 80.8% of patients 1 month after TAVR, (4) all definitions of deterioration in kidney function were associated with a significant increase in 2-year mortality, (5) a deterioration of 10% or more between baseline and steady state eGFR after TAVR was associated with increased mortality in patients with or without baseline CKD, (6) the factor most strongly associated with steady state deterioration in eGFR and CKD resolution after TAVR was periprocedural AKI that did not resolve by discharge, and (7) other factors associated with CKD resolution after TAVR were baseline eGFR, ejection fraction, and the ratio of contrast used to baseline eGFR.

Our results, similar to those published by Beohar et al^7^ and more recently by Cubeddu et al,^8^ draw attention to the interaction between aortic valve stenosis and renal dysfunction. There is growing evidence that the hemodynamic effects of AS can result in a similar association with kidney function as those previously described in patients with congestive heart failure and type 2 CRS.^5^ Also similar to the association between heart failure and kidney function, deteriorating kidney function has been shown to accelerate the progression of AS^13^ and thus can initiate a vicious cycle leading to worsening dysfunction of both organs. Treating the AS through TAVR has the potential to break this cycle and lead to improvements in cardiac as well as kidney function. Patients with AS and CKD secondary to CRS are probably those who will gain the most benefit from TAVR, given the potential for benefits to the kidneys that will be additive to the cardiac benefit related to the treatment of the valvular disease. Considering the potential for improvement in kidney function associated with TAVR, the time course of which is still unknown, it seems probable that steady state rather than periprocedural AKI (which has been the focus of most studies examining the interaction between TAVR and kidney function thus far)^4^ may be more predictive of future prognosis.

To the best of our knowledge, steady state changes in kidney function after TAVR have been examined by only 2 previous studies. In the first, Blair et al^14^ reported on outcomes of 168 patients undergoing TAVR between 2008 and 2014 and found that 1-month deterioration in kidney function after TAVR, but not periprocedural deterioration after TAVR, was associated with worse 1-year survival. Beohar et al^7^ analyzed data from the PARTNER 1 trial and registry and showed that in patients with baseline CKD, deterioration in kidney function after TAVR was associated with increased 1-year mortality. Overall, the design of our study is similar to that of Beohar et al,^7^ as are our results regarding the fraction of patients in whom steady state kidney function deteriorates after TAVR (26% in the overall cohort and 23% in those with baseline CKD compared with 24% in the previous cohort that included only patients with CKD). Similar to Beohar et al,^7^ we found that deterioration in steady state kidney function was associated with increased mortality but that its improvement was not associated with reduced mortality. We believe our results add to the data reported by Beohar et al^7^ in several ways. We included patients with no CKD at baseline and found that changes in kidney function were associated with increased mortality for this population as well, whereas Beohar et al^7^ included only patients with CKD at baseline. Our results suggest the importance of preserving kidney function in patients with no baseline CKD. In addition, our study represented mostly current TAVR practices and patients (40.5% of patients treated during 2017-2019 and only 20.3% of patients treated before 2013; mean [SD] STS score, 5.3% [4.0%]), whereas Beohar et al^7^ studied a cohort representative of an earlier stage in TAVR practice (treated during 2007-2009; median STS score, 10.8% [range, 9.1%-13.3%]). Our cohort included patients treated with balloon-expandable as well as self-expandable devices, whereas Beohar et al^7^ included only patients treated with balloon-expandable devices. All of these factors make our results applicable and relevant to a much larger fraction of current candidates for TAVR. Our follow-up period was longer (2 years compared with 1 year for Beohar et al^7^); we also analyzed data on periprocedural AKI, the association of periprocedural AKI with steady state changes in eGFR after TAVR, and the association of change in CKD status with mortality (which showed a significant benefit for patients receiving TAVR whose CKD status improved after TAVR). Compared with Blair et al,^14^ the fraction of patients with deteriorated kidney function after TAVR was higher (26% vs 12.5%), which may be related to the different definitions of kidney function deterioration (we, like Beohar et al,^7^ used a 10% reduction in eGFR, whereas Blair et al^14^ used a definition of a 0.3mg/dL increase in SCr). As with Beohar et al^7^ compared with Blair et al^14^ (patients treated during 2008-2014; mean [SD] STS score, 9.3% [4.1%]), our cohort was more representative of current TAVR practice, was much larger in terms of sample size (894 compared with 168 patients), and examined longer-term survival (2 years compared with 1 year).

When comparing our results to those reported by Cubeddu et al,^8^ we must first remember several differences in design. Whereas our main focus was on steady state changes in eGFR after TAVR, Cubeddu et al^8^ compared changes in kidney function 7 days postprocedure (whereas the median admission length in their cohort was 5 days), and the fraction of patients with missing SCr levels at follow-up was much higher compared with our cohort (39% vs 18%). Cubeddu et al^8^ examined changes in kidney function according to CKD stage only, whereas we examined percentage changes in eGFR as well as CKD stage. These different definitions are important to note, because in cases when a patient’s baseline eGFR is close to the threshold values separating CKD stages, small changes in eGFR may qualify as a change in CKD stage that would not qualify as improved or deteriorated kidney function under our definition of 10% change in eGFR. Finally, Cubeddu et al^8^ did not examine the association between changes in CKD stage or subsequent mortality 7 days after TAVR. Remembering all these differences, the main results of both studies were consistent. In our cohort, CKD stage remained stable or improved in 80.8% of patients 1 month after TAVR, whereas Cubeddu et al^8^ found that 7 days after TAVR, CKD stage remained stable or improved in 89.2% of patients. In both cohorts, the risk for progression to stage 5 CKD after TAVR was less than 1%. Again, as in Beohar et al,^7^ our cohort was more representative of current TAVR practice (in Cubeddu et al,^8^ patients were treated during 2007-2014, 72% were at high or inoperable risk, and all were treated with balloon-expandable valves).

Although there is strong evidence from a meta-analysis of randomized trials that short-term, mild-to-moderate increases in SCr are not associated with meaningful midterm clinical outcomes (CKD/mortality),^15^ our results, as well as those published by Beohar et al^7^ and Cubeddu et al^8^ (albeit all observational in nature), did find short-term to midterm changes in SCr to be associated with midterm mortality. This difference may be due to bias and confounding resulting from the observational nature of these studies, but it may also be related to the different patient populations and the nature of the interventions. First, although the randomized trials included in the cited meta-analysis included patients with a mean age of 65 years, the mean age of patients receiving TAVR is approximately 80 years, which is expected to have a bearing on the patients’ comorbidity burden (expected to be more severe in the TAVR population) and would therefore make patients receiving TAVR more vulnerable to even mild or moderate changes in kidney function than younger patients with a smaller overall comorbidity burden. Second, most trials included in the meta-analysis examined pharmacologic interventions and invasive procedures that involved exposure to contrast material, which were not common in TAVR, therefore limiting the applicability of the study conclusions to the TAVR population.

Our results have 2 main implications for clinical practice. First, they provide reassurance as to the safety of TAVR in terms of kidney outcomes, both in the overall population as well as in those with AS and CKD (a subgroup of patients who may be hesitant to undergo TAVR, fearing a detrimental effect on kidney function). Second, they underscore the importance of deterioration in kidney function after TAVR, either periprocedural or at steady state, and stress the need to prevent kidney injury in patients undergoing TAVR regardless of baseline renal function. Finally, our findings demonstrate the potential for TAVR to improve kidney function and showed that this improvement may be associated with an additive benefit in patients whose CKD status resolved after TAVR. This added benefit was probably reserved mainly for the patients whose kidney dysfunction was caused predominantly by type 2 CRS.

The ability to identify these patients is valuable. However, like in Beohar et al,^7^ our study found that predicting the kidney response to TAVR, in terms of function deterioration or improvement and in terms of CKD resolution, was challenging, which was not surprising given the multifactorial causes of kidney dysfunction at baseline. Only female sex and baseline eGFR less than 60 mL/min/1.73 m^2^ were found to be associated with improvement in kidney function after TAVR. Because women undergoing TAVR tend to have a lesser comorbidity burden compared with men,^16^ it is reasonable to assume that CRS was more prevalent as a cause for kidney dysfunction in women as compared with men, which would explain their higher likelihood for improvement in kidney function after TAVR.

When examining the factors found to be associated with deterioration in kidney function or change in CKD status after TAVR, our results highlight the importance of minimizing contrast exposure and prevention of AKI. The strongest association for both outcomes was with AKI not resolving by discharge, and an association was found with the contrast-eGFR ratio. The contrast-eGFR ratio is well established as a predictor of AKI for patients undergoing coronary procedures,^17^ and it has also been shown to be associated with worse kidney outcomes in patients with transplanted kidneys who undergo TAVR.^18^ Although other factors associated with new CKD status or deterioration in kidney function after TAVR cannot be modified (such as STS score, age, and baseline eGFR), minimizing contrast exposure can be promoted by several means: lower rates of injections, using ultrasound guidance to avoid the need for angiography to guide the femoral puncture, fusion imaging to replace aortograms for placement of the device, and echocardiographic assessment of perivalvular leaks instead of using postimplantation aortogram. All these measures can be used at the discretion of the operators if there is sufficient awareness of the need to minimize contrast exposure and the potential harm related to AKI. Other measures to prevent AKI focus on adequate preprocedural hydration, and dedicated intraprocedural-matched hydration-diuresis protocols are mainly supported by data from coronary procedures^19,20^ that have either not been examined or have not yet been shown to be beneficial^21^ in patients receiving TAVR.

### Strengths and Limitations

Our study has several strengths. To our knowledge, it is the first to examine the association of eGFR with mortality after TAVR in relation to periprocedural as well as steady state changes. The patient population was representative of real-world patients undergoing TAVR according to current clinical practice, and follow-up duration was longer compared with previous studies on this issue.

Our study also has several limitations. Its observational and retrospective design renders it prone to residual bias and confounding, even after using multivariate adjusted models. The study is based on data from a single center. The SCr level at 1 month after TAVR was missing in more than 10% of the cohort, and we could not assess the association of concomitant PCI during TAVR with either kidney function or survival after TAVR, because our practice does not perform concomitant PCI during TAVR procedures. Our definition of AKI was a modification of that used by the VARC 2 criteria, and our timing of assessment for kidney function change was different from that recommended by the Kidney Disease–Improving Global Outcomes criteria for AKI resolution (1 month compared with 3 months). We examined steady state renal function only at 1 month after TAVR and therefore cannot examine the optimal timing to assess changes in steady state kidney function after TAVR. Our data covered a period of more than 11 years, during which many changes and advances in the practice of TAVR occurred in terms of the procedure itself as well as patient selection and postprocedural care; therefore, not all patients included in the study could be considered to represent current clinical practice.

## Conclusions

In this cohort study, steady state renal outcomes after TAVR were reassuring, with more than 80% of patients showing stable or improved kidney function 1 month after the procedure. Improvement in kidney function had a favorable association with 2-year mortality, whereas deterioration in kidney function was associated with increased mortality regardless of baseline CKD status. Future studies should focus on identifying the role of CRS as a main cause of CKD in the AS population in order to improve patient selection and optimize outcomes after TAVR.

## Notes

Supplement. **eFigure 1.** Cohort Selection Process for Steady State and Periprocedural Analysis **eFigure 2.** Steady State Change in Renal Function According to Baseline eGFR **eFigure 3.** Distribution of State Change in Renal Function After TAVR **eFigure 4.** Steady-State eGFR According to Baseline eGFR **eFigure 5.** Kaplan-Meier Curves for 2-Year Mortality According to Steady-State Changes in Renal Function^a^ **eFigure 6.** ROC Curves for Prediction Models for Steady-State Renal Function Deterioration (left) and CKD Status Resolution After TAVR **eTable 1.** Periprocedural Complications According to Steady-State Change in eGFR Category **eTable 2.** Periprocedural Complications According to AKI Category **eTable 3.** Predictors of Periprocedural/Steady-State Changes in eGFR Function After TAVI
