# Supplementary material for: Change in Kidney Function and 2-Year Mortality After Transcatheter Aortic Valve Replacement
Source: JAMA Netw Open. 2021 Mar 26;4(3):e213296. doi: 10.1001/jamanetworkopen.2021.3296 (PMC7998079; doi:10.1001/jamanetworkopen.2021.3296)
Supplement: Supplement. — eFigure 1. Cohort Selection Process for Steady State and Periprocedural Analysis eFigure 2. Steady State Change in Renal Function According to Baseline eGFR eFigure 3. Distribution of State Change in Renal Function After TAVR eFigure 4. Steady-State eGFR According to Baseline eGFR eFigure 5. Kaplan-Meier Curves for 2-Year Mortality According to Steady-State Changes in Renal Functiona eFigure 6. ROC Curves for Prediction Models for Steady-State Renal Function Deterioration (left) and CKD Status Resolution After TAVR eTable 1. Periprocedural Complications According to Steady-State Change in eGFR Category eTable 2. Periprocedural Complications According to AKI Category eTable 3. Predictors of Periprocedural/Steady-State Changes in eGFR Function After TAVI [file jamanetwopen-e213296-s001.pdf]

Supplementary Online Content

Witberg G, Steinmetz T, Landes U, et al. Change in kidney function and 2-year mortality after transcatheter aortic valve replacement. *JAMA Netw Open*. 2021;4(3):e213296. doi:10.1001/jamanetworkopen.2021.3296

eFigure 1. Cohort Selection Process for Steady State and Periprocedural Analysis

eFigure 2. Steady-State Change in Renal Function According to Baseline eGFR

eFigure 3. Distribution of State Change in Renal Function After TAVR

eFigure 4. Steady-State eGFR According to Baseline eGFR

eFigure 5. Kaplan-Meier Curves for 2-Year Mortality According to Steady-State Changes in Renal Function[a]

eFigure 6. ROC Curves for Prediction Models for Steady-State Renal Function Deterioration (left) and CKD Status Resolution After TAVR

eTable 1. Periprocedural Complications According to Steady-State Change in eGFR Category

eTable 2. Periprocedural Complications According to AKI Category

eTable 3. Predictors of Periprocedural/Steady-State Changes in eGFR Function After TAVI

This supplementary material has been provided by the authors to give readers additional information about their work.

© 2021 Witberg G et al. *JAMA Network Open.*

eFigure 1. Cohort Selection Process for Steady State and Periprocedural Analysis

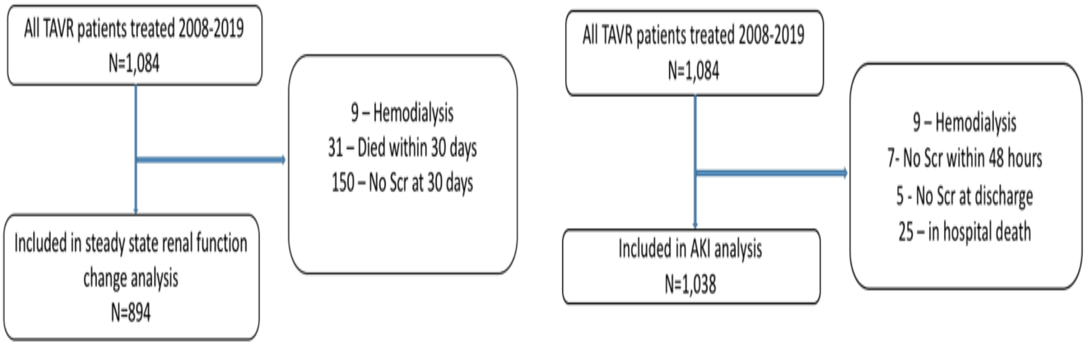

eFigure 2. Steady-State Change in Renal Function According to Baseline eGFR

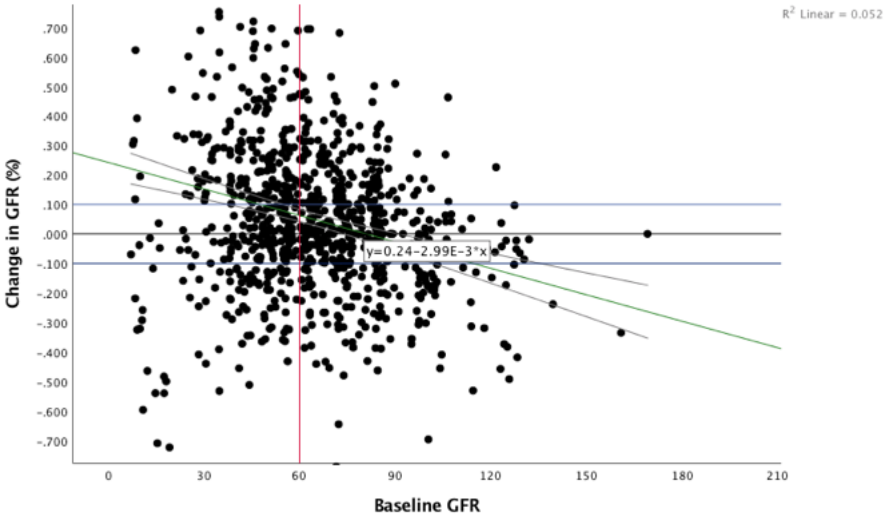

© 2021 Witberg G et al. *JAMA Network Open.*

eFigure 3. Distribution of State Change in Renal Function After TAVR

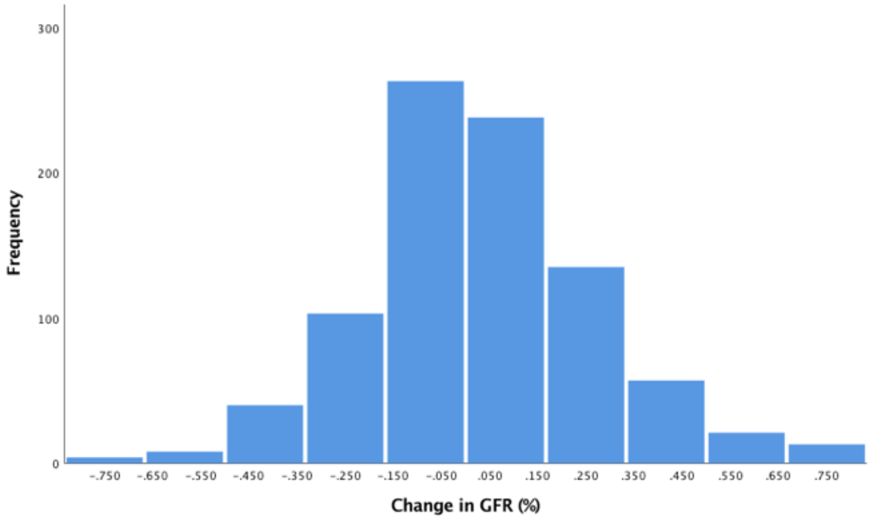

© 2021 Witberg G et al. *JAMA Network Open.*

eFigure 4. Steady-State eGFR According to Baseline eGFR

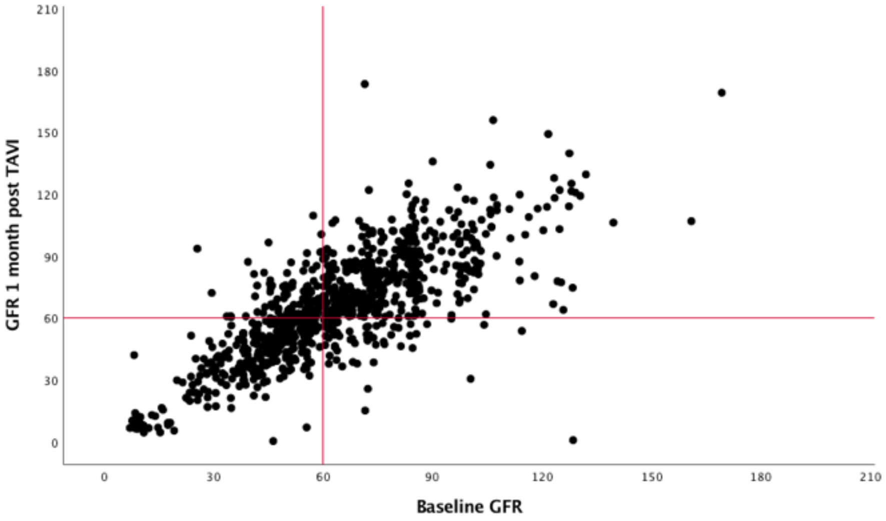

© 2021 Witberg G et al. *JAMA Network Open.*

eFigure 5. Kaplan-Meier Curves for 2-Year Mortality According to Steady-State Changes in Renal Function[a]

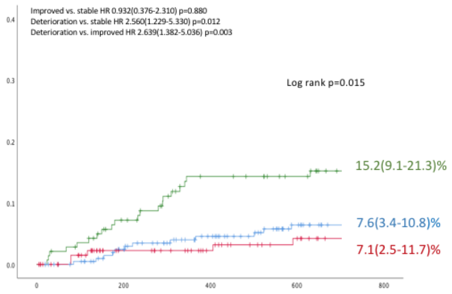

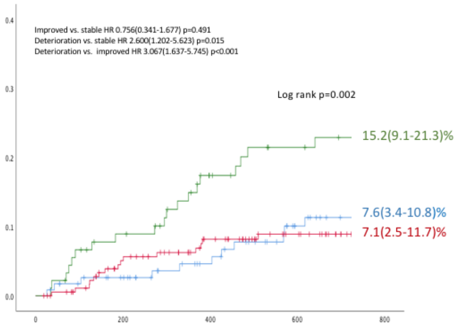

---

[a]Baseline eGFR ≥ 60 mL/min (left) and eGFR < 60 mL/min (right).

© 2021 Witberg G et al. *JAMA Network Open.*

eFigure 6. ROC Curves for Prediction Models for Steady-State Renal Function Deterioration (left) and CKD Status Resolution After TAVR

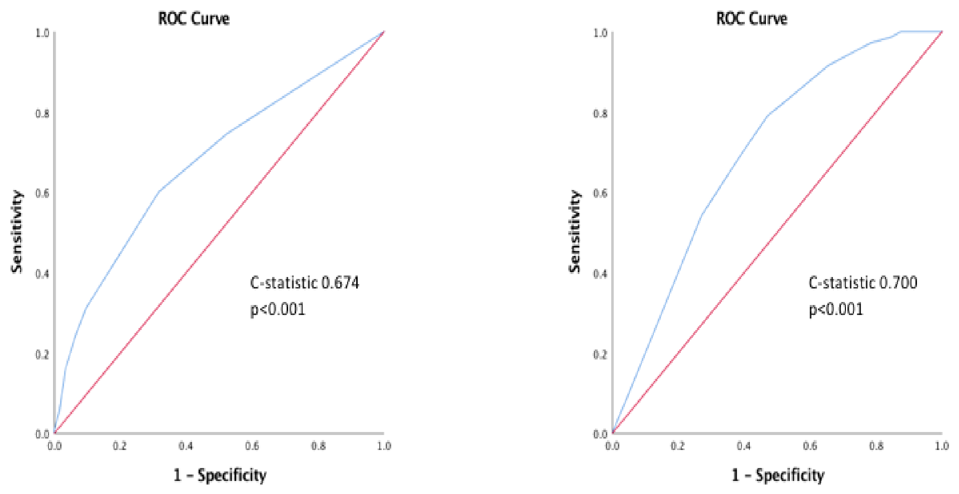

© 2021 Witberg G et al. *JAMA Network Open.*

eTable 1. Periprocedural Complications According to Steady-State Change in eGFR Category

	Stable GFR N=332	GFR improvement N=329	GFR deterioration N=233	P value
Vascular complications				0.312
Minor	53(16.0%)	54(16.5%)	40(12.9%)	
Major	5(1.5%)	12(3.7%)	8(3.4%)	
Bleeding complications				0.17
Minor	8(1.4%)	14(4.3%)	6(2.6%)	
Major	4(1.2%)	7(2.1%)	3(1.3%)	
Life threatening	0(0%)	0(0%)	2(0.9%)	
Pacemaker implantation	41(12.3%)	42(12.8%)	30(12.9%)	0.98

eGFR=estimated glomerular filtration rate

eTable 2. Periprocedural Complications According to AKI Category

	No AKI N=921	AKI N=115	P value
Vascular complications			<0.01
Minor	143(15.5%)	17(14.7%)	
Major	15(1.6%)	14(12.1%)	
Bleeding complications			<0.01
Minor	9(1.0%)	3(2.6%)	
Major	16(1.7%)	4(3.5%)	
Life threatening	1(0.1%)	3(2.6%)	
Pacemaker implantation	111(12.1%)	20(17.3%)	0.14

AKI=acute kidney injury

© 2021 Witberg G et al. *JAMA Network Open.*

eTable 3. Predictors of Periprocedural/Steady State-Changes in eGFR

Function After TAVI

	OR	95% CI	P value
**A AKI resolution**			
No frailty	4.77	1.44-15.79	0.01
Age<80	8.20	2.70-14.64	<0.01
**B Steady state renal function deterioration**			
STS	1.06	1.02-1.09	0.01
STS>6	1.43	1.03-1.98	0.03
GFR (per 1ml/min)	1.01	1.01-1.02	<0.01
GFR>75 ml/min	1.92	1.40-2.62	<0.01
AKI resolved at discharge	2.82	1.70-4.70	<0.01
AKI not resolved at discharge	7.32	4.53-11.42	<0.01
**C Steady state renal function improvement**			
Female	1.27	1.03-1.61	0.04
GFR<60	2.28	1.73-3.01	<0.01
**D Steady state CKD resolution**			
STS	0.87	0.81-0.94	<0.01
STS<6	1.81	1.10-2.97	0.02
LVEF	1.04	1.01-1.08	0.02
LVEF>50%	2.15	1.11-4.18	0.02
GFR	1.12	1.08-1.16	<0.01
GFR>45 ml/min	3.13	1.83-6.92	<0.01
Contrast/GFR	1.40	1.14-1.72	<0.01
Contrast/GFR<3.5	4.03	2.11-7.69	<0.01
No AKI at discharge	4.62	2.15-6.44	<0.01

AKI=acute kidney injury GFR=glomerular filtration rate LVEF=left ventricle ejection fraction STS=society of thoracic surgeons

© 2021 Witberg G et al. *JAMA Network Open.*
